# Advances in the study of FOXQ1: biological functions and mechanisms

**DOI:** 10.3389/fonc.2025.1650022

**Published:** 2025-11-19

**Authors:** Xiaojian Feng, Ling Zhang, Peiyao Shi, Yiping Hu

**Affiliations:** 1 Southern University of Science and Technology Yantian Hospital, Shenzhen, Guangdong, China; 2 Shenzhen Peking University-The Hong Kong University of Science and Technology Medical Center, Shenzhen, China; 3 Peking University Shenzhen Hospital, Shenzhen, China

**Keywords:** FOXQ1, forkhead box, epithelial-mesenchymal transition, cancer, metastasis

## Abstract

Forkhead box Q1 (FOXQ1) is a member of the Forkhead box gene family and an important transcription factor closely associated with several human diseases, especially tumorigenesis and tumor progression. This review aims to explore advances in the study of the biological functions of FOXQ1 in several tumors, such as colorectal cancer, breast cancer, esophageal cancer, nasopharyngeal cancer, lung cancer, hepatocellular cancer, pancreatic cancer, gastric cancer, melanoma, bone-related disease, immune and inflammatory disease, regulatory factors of FOXQ1 expression, and mechanism of tissue-specific function. FOXQ1 influences the pathological progression of these diseases through different targets genes and signaling pathways, which we also review in detail. In conclusion, more and more FOXQ1 applications and different pathologic mechanisms are bound to be reported in future studies.

## Introduction

The forkhead box (FOX) family is present in wide-ranging organisms, from yeast to humans. Based on the homology of DNA−binding domains, more than 100 FOX family members have been reported and been divided into 19 subfamilies, denoted as FOXA-S, and more than fifty types of FOX proteins were encoded by the human genome (1–4). The FOX family is characterized by a conserved winged-helix DNA-binding domain. As transcriptional regulators, FOX proteins participate in diverse biological processes and are closely linked to tumor initiation and progression (5–7).

**Figure 1:**
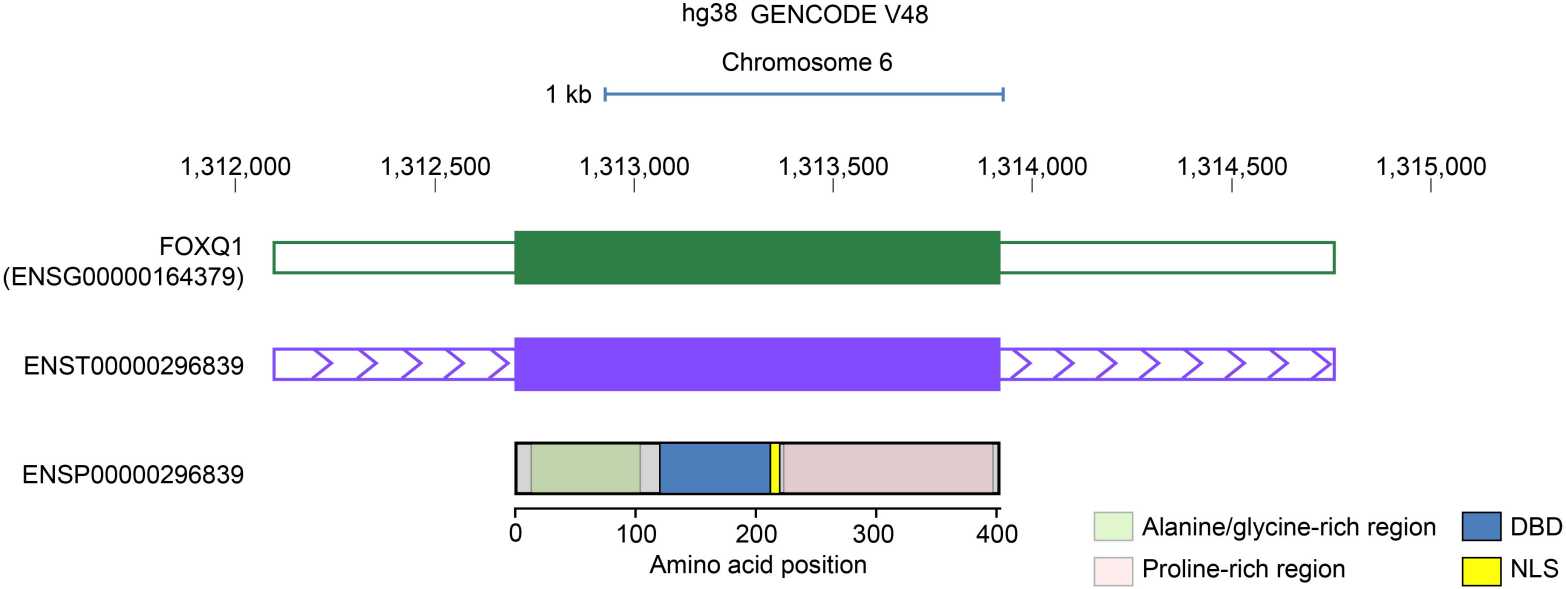
Location and structure of the gene coordinate of human FOXQ1: Scale bar (blue ruler at top). The prediction of possible transcription activation domains was done in ADpred (104). FOXQ1 (ENSG 00000164379): the green box indicates the gene’s Ensembl ID and coordinate location. ENSTO0000296839: the purple box indicates the gene transcript ID and coordinate position. The DNA-binding domain (DBD), the nuclear localization sequence (NLS), Alanine/glycine-rich region and proline-rich region.

The FOX family not only has numerous members and extensive functions involving multiple biological processes (8–10). FOXQ1 as one of the main members of the FOX family that has also been studied by numbers scholars. The human FOXQ1 gene is located at 6p25.3 and comprises 2661 base pairs, encoding 403 amino acids (aas). The FOXQ1 protein is divided into three domains: The alanine and glycine enrichment region, the proline-enrichment region and the forkhead box domain (FHD) or winged-helix domain. The N-terminal alanine/glycine-rich domain (at aa 13-103) and the C-terminal proline-rich domain (at aa 221-397) were associated with the transcriptional regulatory activity of the protein (2) (**Figure 1**). It is related to the occurrence and development of various human diseases, especially various tumors (1, 5, 11). This article provides a review of the research progress on the role, pathogenic mechanism, and prevention and treatment of FOXQ1 in various tumors and immune diseases.

## Disease pathogenesis roles of FOXQ1

FOXQ1 is a transcription factor that plays a key pathological role in various diseases. This section reviews its pathological mechanisms in different cancers, bone-related diseases, immune and inflammatory diseases.

## FOXQ1 and colorectal cancer

Colorectal cancer (CRC) is a major health problem, with multiple tissue pathological processes and the pathological mechanisms that are currently not fully clear (12–14). Jia Yun Liu et al. (15) found that FOXQ1 is markedly overexpressed in CRC cells and CRC samples from clinical patients. FOXQ1 promotes cancer aggressiveness, such as cell proliferation, migration, and invasion *in vitro*, as well as growth *in vivo*, through the activation of the FAK/PI3K/AKT signaling pathway. FOXQ1 can promote the invasion and metastasis of CRC through the heparin-binding epidermal growth factor (HB-EGF)/EGFR pathway (16). TGF-β induces the expression of FOXQ1, inducing the tumor characteristic in CRC cells by modulating the Wnt/β-catenin pathway (17). Mediated sirtuin 1(SIRT1) expression and promoting β-catenin nuclear translocation are the key functions of FOXQ1in the therapy of CRC (18). Zhihu Liu et al. (19) demonstrated that miR-106a can improve the sensitivity of CRC cells to oxaliplatin through direct downregulation of FOXQ1expression. miR-378a acts as an inhibitor by preventing the abnormal activation of FOXQ1-cMYC axis signaling to improve the tumor characteristics of CRC (20). miR-342 is a tumor suppressive miRNA targeting the regulation of FOXQ1 biological function and thus affecting CRC development and clarified FOXQ1 as a valuable prognostic marker for CRC. FOXQ1 expression was validated in two large screening cohorts (n=550) and an independent clinical validation cohort (n=134), demonstrating that high FOXQ1 expression is an independent prognostic factor in colorectal cancer patients (21).

Based on the above reports, FOXQ1 plays a crucial role in the occurrence and development of CRC, it is also regulated by other factors. The long non-coding RNA (lncRNA) RP9 pseudogene (RP9P), by mediating the activity of miR-133a-3p/FOXQ1 axis regulates CRC cell tumorigenesis (22). LINC00543 remolds the tumor-microenvironment in CRC and enhances the epithelial-mesenchymal transition (EMT) of CRC cells through the pre-miR-506-3p/FOXQ1 axis (23). Similarly, the JAK2/STAT3/miR-506-3p/FOXQ1 signaling pathway plays a key role in the interaction between immune cells and cancer cells in the CRC pathological microenvironment (24). Hui Tang, et al. found that FOXQ1 can inhibit angiogenesis and reduce macrophage recruitment and may thus serve as a therapeutic target in CRC (25).

## FOXQ1 and breast cancer

Breast cancer is the most common global malignancy and the leading cause of cancer deaths, especially in female patients. Despite some progress in its treatment, metastatic tumors remain incurable and require new therapeutic targets and strategies (26, 27). Some issues of concern include the regular supervision of survivors of cancer, the management of treatment side effects, the implementation of a normal lifestyle and the regulation of psychological factors (28, 29). Fahed A Elian et al. (30) found that FOXQ1 is differentially expressed in breast cancer subtypes and indicates a lower overall survival rate in breast cancer patients with low expression of FOXQ1. FOXQ1 protein expression is significantly higher in triple-negative breast cancer samples than in normal mammary tissues, so it is a novel target of breast cancer stem cells’ inhibition by diallyl trisulfide (31).

The RNA-binding protein Hu antigen R (HuR) is upregulated and is a direct target that regulates FOXQ1 in human breast cancer. These two factors’ interactions regulate the invasion and metastasis of breast cancer (32). By activating of the FGFR1-MEK-ERK2-c-FOS signaling axis, FOXQ1 expression is upregulated, FOXQ1 is an essential factor mediating the FGFR1 signaling and promotes breast cancer worsening (33). Other studies have found that FOXQ1 increases complex I-linked oxidative phosphorylation and contributes to the oncogenic role in human breast cancer cells through the direct transcriptional regulation of NDUFS1 and NDUFV1 (34). FOXQ1 is a therapy target of a small- molecule drug, Withaferin A (WA), derived from a medicinal plant (*Withania somnifer*). It inhibits breast cancer cell proliferation and cell migration (35). Additionally, miR-937 acts as an inhibitor to directly target and regulate FOXQ1, thereby limiting breast cancer cell expansion and cancer progression (36).

Haijun Zhang et al. (37) found that FOXQ1 expression is regulated by TGF-β1, and its repressed expression of E-cadherin mediates EMT by recruiting the mixed-lineage leukemia/histone methyltransferase 2 (MLL/KMT2) histone methyltransferase complex as a transcriptional coactivator (38). Additionally, the HuR inhibitors KH-3 can break the mRNA interaction of HuR-FOXQ1 axles, leading to the inhibition of breast cancer invasion (32). FOXQ1 directly transcriptionally regulates interleukin (IL)-8, IL-1α, and vascular endothelial growth factor (VEGF) to affect the pathological mechanism of human breast cancer cells (31, 39). Similarly, Nuclear isoform of RAPH1 (named RAPH1-i3) interacts with FOXQ1 and can promote breast cancer progression and radio-resistance (40). FOXQ1, as a transcription regulator, mediates the biological function of multiple genes in breast cancer, so it is a key target for chemical and biological targeted therapy (41–43). However, benzyl isothiocyanate as a breast cancer chemoprevention agent regulates FOXQ1 expression and mediates the inhibition of EMT in human breast cancer cells (44).

## FOXQ1 and esophageal cancer

Esophageal squamous cell carcinoma (ESCC) is one of the most malignant cancers and lacks unified standards for diagnosis and treatment (45, 46). FOXQ1’s abnormal upregulation of expression in human ESCC cells and knockdown of FOXQ1 can restrain the tumor characteristics of different ESCC cells in a mouse xenograft model *in vivo (*47). However, FOXQ1 acts an oncogene in human ESCC cells by negatively regulating CDH1 to promote ESCC cell proliferation and metastasis (48). The biological function of FOXQ1 is still being explored in ESCC, and current research is not very extensive.

## FOXQ1 and nasopharyngeal carcinoma

FOXQ1 promotes EGFR expression at mRNA and protein levels, mediates the EGFR signaling activity, and increases the metastasis of nasopharyngeal carcinoma (NPC) by inducing vasculogenic mimicry (49). miR-342-3p and miR-124 targets the regulation of FOXQ1 expression by mediating NPC cell growth and invasion (50, 51). Moreover, lncRNA 00667 acts an oncogene by promoting NPC cell growth through competitive targeting and binding to miR-4319 to upregulate FOXQ1 expression (52). Circular RNA CRIM1 inhibits the suppressive effects of miR-422a on its target gene FOXQ1 and leads to its expression upregulation, resulting in NPC metastasis, EMT, and docetaxel chemoresistance (53).

## FOXQ1 and lung cancer

FOXQ1 is highly expressed in non-small cell lung cancer (NSCLC) tissue samples from patients and can be potentially used as an EMT marker in NSCLC (54, 55). Further survival analysis surfaced high FOXQ1 expression as an independent prognostic factor (54). Likewise, FOXQ1 is over-expressed in NSCLC cancer tissue compared with adjacent tissue. FOXQ1 expression was increased in tumor tissue (61.3% high expression and 38.7% low expression) compared with paired adjacent tissue (37.8% high expression and 62.2% low expression) (*P <* 0.001). It is associated with the malignant features of tumors. High FOXQ1 expression is an independent risk factor for disease-free survival and overall survival in patients with NSCLC, indicating the potential prognostic value of FOXQ1 for NSCLC (56). miR-133 also downregulates FOXQ1 expression to mediate the EMT and antagonizes lung cancer tumorigenesis (57).

## FOXQ1 and hepatocellular carcinoma

The high expression of FOXQ1 in hepatocellular carcinoma (HCC) cells may be related to its biological function as a therapeutic target for HCC. Liposomal clodronate combined with cisplatin or sorafenib inhibits FOXQ1 expression in HCC cells and restrains their proliferation, migration, and invasion (58, 59). Certainly, these studies have demonstrated that high FOXQ1 expression is an independent factor in the prognosis of HCC (59). miR-4319 is a post-transcriptional regulator that directly dampens the expression of FOXQ1 to decrease cell proliferation, inhibit EMT, accelerate apoptosis, and prevent cancer stemness in HCC (60). Additionally, FOXQ1/NDRG1 (N-myc downstream-regulated gene 1) axis plays a key role between HCC and cancer-associated fibroblast (CAF) crosstalk (61). FOXQ1 further induces sex determining region Y-box 12 (Sox12) overexpression and promotes HCC invasion and metastasis through the transcriptional activation of Twost1 and fibroblast growth factor binding protein 1 (FGFBP1). Further results showed that patients with positive FoxQ1/Sox12 expression had a poorer prognosis (62).

## FOXQ1 and pancreatic cancer

Zhan HX, et al. (63) found that FOXQ1 expression is negatively associated with the overall survival of PC patients. FOXQ1 overexpression in PC stem-like cells and inhibition of FOXQ1 attenuates tumor formation, growth, and so on. Thus, it may be a novel therapy for achieving a better treatment outcome of PC (64). FOXQ1 via promotes the transcription of Lactate dehydrogenase A (LDHA) and increases its expression in pancreatic cancer (PC). It then activates aerobic glycolysis to stimulate PC cell proliferation, tumor stemness, invasion, metastasis, and so on (65).

## FOXQ1 and gastric cancer

A high expression of FOXQ1 is involved in the acquisition of the mesenchymal phenotype of GC cells. The subsequent activation of the expression of the Snail signaling pathway is essential for EMT induction. Thus, FOXQ1 is a potential prognostic marker and is therapeutic for patients with GC (66, 67). miR-96-5p inhibits the protein expression of FOXQ1 suppresses the proliferation, migration, and EMT of gastric cancer cells (68). miR-345 inhibits metastasis *in vitro* and *in vivo*, as well as the EMT of GC cells, via the targeted regulation of FOXQ1 expression (69). miR-519 was abnormally low expression and involve to GC tumor characteristics and regulation the biological behavior of GC cells via direct targeting FOXQ1 (70). Accordingly, miR-1271 acts as a novel tumor suppressor that inhibits the proliferation, invasion, and EMT of the GC cells by downregulating the expression of FOXQ1 (71). Similarly, tumor-associated macrophages are play important roles in the tumor microenvironment, such as promoting EMT, invasion, and migration in GC cells. These biological processes may be mediated by FOXQ1 (72).

## FOXQ1 and melanoma

Despite rapid advances in tumor diagnostics and pathogenic molecular mechanisms in recent years, the early clinical diagnosis and histopathology of melanoma remain inadequate (73, 74). Archis Bagati, et al. (75) found a novel mechanism involving the opposite roles of FOXQ1 in the regulation of N-cadherin (CDH2) gene, invasion and metastasis in melanoma versus carcinoma cells, FOXQ1 through interacts with nuclear β-catenin and TLE and altering the levels of these two proteins suppressor melanomas. FOXQ1 also directly regulates the transcriptional activation of the MITF gene (a melanocytic lineage-specific regulator of differentiation) and thus promotes the differentiation of normal and transformed melanocytic cells (76).

## FOXQ1 and bone-related diseases

Abnormal mesenchymal stem cells (MSCs) osteogenic differentiation abnormal is play very important role in multiple bone diseases (77, 78). Clinical findings show a higher fracture risk of osteoporosis (OP) patients complicated with type II diabetes mellitus (T2DM) compared with the non-T2DM patients. MSCs from T2DM patients also show a weaker osteogenic potent. Upon investigation, FOXQ1 downregulation causes a decline in osteogenic potential of T2DM-BSMC. This mechanism may be the key to research on the treatment of diabetic OP (79). FOXQ1 acts as a mediator that regulates the activities of Wnt/beta-catenin signaling by binding with ANXA2 to promote osteogenic differentiation of MSCs (80). FOXQ1 reportedly inhibits Osteoarthritis (OA) progression by downregulating pyroptosis induced by NLR family pyrin domain containing 3 (NLRP3) (81).

## FOXQ1 and immune and inflammatory diseases

As one of the early and extensively studied forkhead genes, the biological structure and function of FOXQ1 have been relatively thoroughly researched (82, 83). We have reviewed the biological functions and pathogenic mechanisms of FOXQ1 in multiple malignant tumors, so exploring it’s necessary to explore its precise function and mechanism in the immune and inflammation system is necessary. High FOXQ1 expression in natural killer/T-cell lymphoma is correlated with cell proliferation, growth, and apoptosis induction. These functions affect multiple pathological processes, such as high Ann Arbor stage, bone marrow involvement, poor prognosis, and inhibition of the shh signaling pathway (84). Similarly, FOXQ1 is highly expressed and directly regulates the inhibition of neurexins 3 (NRXN3) expression, thereby promoting to proliferation and migration in glioma cells (85).

Recent research reports USP10 deubiquitinating regulates FOXQ1, which plays a protective role in sepsis-induced acute kidney injury (S-AKI) by inducing inflammation and apoptosis (86). FOXQ1 also reportedly binds directly to inhibit prostaglandin-endoperoxide synthase 2 (PTGS2), and cyclin-dependent kinase 5 (CDK5) to promote apoptosis and inflammation while inhibiting neurite outgrowth in Alzheimer Disease (87). Xiaohui Ma et al. found that miR-125b directly targets FOXQ1 by regulating the neuronal cell apoptosis and phosphorylation of Tau (88). In chronic inflammatory atopic dermatitis pathology, FOXQ1 stimulates monocyte motility and increases pro-inflammatory potential. It can also induce monocyte migration toward MCP-1, which is essential for monocyte influx into inflammatory sites (89). Another study has found that FOXQ1 may influence the NK-cell and alloimmune cytotoxic T-cell functions in the differentiation and development of hair shaft (90).

Based on the above insights into FOXQ1’s role across various diseases, we propose distinct approaches for the clinical translation of FOXQ1. Indirect Suppression of FOXQ1: Indirect inhibition strategies downregulate FOXQ1 expression through upstream pathway inhibitors (such as those targeting Wnt/β-catenin or TGF-β signaling pathways) or miRNA mimics (37). Hypothetical Direct FOXQ1 Targeting: Directly targeting the transcription factor FOXQ1 itself remains a medium-to-long-term research direction, with the core challenge being the widespread “druggability” issue inherent to transcription factors (91). Additionally, we summarized the expression patterns of FOXQ1 in the aforementioned diseases, along with its corresponding direct or indirect targets and various biological functions (**Table 1**).

**Table 1:** Expression of FOXQ1, interacting gene targets and biological functions.

Tumor type	Expression of FOXQ1	Interacting target gene	Functions	Refs
Colorectal cancer	up	EGFR, TGF-β, SIRT1, miR-106a, miR-378a, miR-342, miR-133a-3p, miR-506-3P, CCL2	metastasis and invasion, angiogenesis, fibroblast marker	(16–25)
Breast cancer	up	TGF-β1, E-cadherin, N-cadherin, RAPH1, FGFR1, KH-3, IL-8, IL-1α, VEGF, NDUFS1, NDUFV1, miR-937	invasion, EMT, radioresistance, metastasis	(31–43)
Esophageal cancer	up	CDH1	proliferation and metastasis, pro- Inflammatory	(47, 48)
Nasopharyngeal Cancer	up	miR-422a, miR-124, EGFR, miR-4319, miR-342-3p	metastasis/docetaxel, chemoresistance, migration and invasion	(49–53)
Lung cancer	up	miR-133	EMT	(57)
Hepatocellular Cancer	up	miR-4319, Sox12, FGFBP1, NDRG1	proliferation, migration and invasion	(60–62)
Pancreatic cancer	up	LDHA	proliferation, tumor stemness, invasion and metastasis, aggressive behavior	(63, 65)
Gastric cancer	up	miR-96-5p, miR-345, miR- 519, miR-1271,	EMT, metastasis and proliferation	(68–72)
Melanoma	down	CDH2, MITF, β-catenin, N-cadherin, TLE-proteins,	EMT, invasion and metastasis, melanocytic cells differentiation	(75, 76)
Bone related disease	up or down	T2DM, Annexin A2, RUNX2, COL1A1, NLRP3	osteogenic differentiation	(79–81)
Immune and Inflammatory disease	up	MCP-1, NRXN3, USP10, CDK5, miR-125b, PTGS2	cell proliferation, apoptosis, chronic inflammatory disorders	(84–90)

EGFR, epidermal growth factor receptor; TGF-β, transforming growth factor-beta; SIRT1, sirtuin 1; CCL2, C-C motif ligand 2; PAPH1, named RAPH1-i3; FGFR1, fibroblast growth factor receptor1; VEGF, vascular endothelial growth factor; KH-3, HuR inhibitors KH-3; NDUFS1, ubiquinone oxidoreductase core subunit S1; NDUFV1, ubiquinone oxidoreductase core subunit V1; CDH1, E-cadherin; FGFBP1, fibroblast growth factor binding protein 1; NDRG1, N-myc downstream-regulated gene 1; LDHA, Lactate dehydrogenase A; T2DM, type II diabetes mellitus; RUNX2, Runt-related transcription factor 2; COL1A1, collagen type I alpha 1; NLRP3, NLR family pyrin domain containing 3; MCP-1, Monocyte chemoattractant protein-1; NRXN3, inhibition of neurexins 3; USP10, ubiquitin-specific peptidase 10; CDK5, cyclin-dependent kinase 5; PTGS2, prostaglandin-endoperoxide synthase 2.

## Regulatory factors of FOXQ1 expression

Many factors have been shown to regulate FOXQ1 expression in different human diseases. Here we focus on the regulation at the transcriptional levels, post-transcriptional levels and inhibitors (**Table 2**).

**Table 2:** Regulatory factors of FOXQ1 expression.

Levels of regulation	Regulatory factors	Disease type	Refs.
Transcriptional	NAC1 Wnt/β-catenin signaling pathway TGF-/Smads signaling pathway	Ovarian Cancer CRC CRC	(10, 17, 92, 93)
Post-transcriptional	miRNAs (miR-124, miR-422a, miR-506, miR-1271) Long noncoding RNA and circular RNAs (LncRNA TUG1, circCD2AP) Ubiquitination and deubiquitination (USP21, USP10) Acetylation and deacetylation (ORF45, P300) Methylation	NPC, GC, HCC Bladder Cancer Lytic Infection CRC Mastocytosis	(71, 86, 94–101)
Inhibitors	Indirect target: HuR, FGFR	Breast Cancer, NPC	(32, 49)

NAC1, nucleus accumbens-associated protein 1; USP21, ubiquitin-specific peptidase 21; USP10, ubiquitin-specific peptidase 10.

Transcriptional regulation of FOXQ1: A study reported that nucleus accumbens-associated protein 1 (NAC1) transcriptionally regulates FOXQ1 expression (92). Additionally, FOXQ1 is regulated as a downstream gene of some classical signaling pathways by members of this pathway, such as glycogen synthase kinase 3, an inhibitor of Wnt/β-catenin, activates FOXQ1 expression in solid tumors (10). Similarly, several studies have also found that FOXQ1 expression is regulated by the TGF-/Smads pathway. The mechanism remains unclear, but CRC cells treated with TGF for 3 days significantly upregulate both mRNA and protein levels of FOXQ1. Interestingly, further investigation of these cells indicated alterations in the Wnt pathway, including vascular endothelial growth factor (VEGF)-A, matrix metalloproteinase 2, vimentin, N-cadherin and E-cadherin, these results indicate that FOXQ1 provides crosstalk between the Wnt and TGF-β signaling pathways (17, 93).

**Figure 2:**
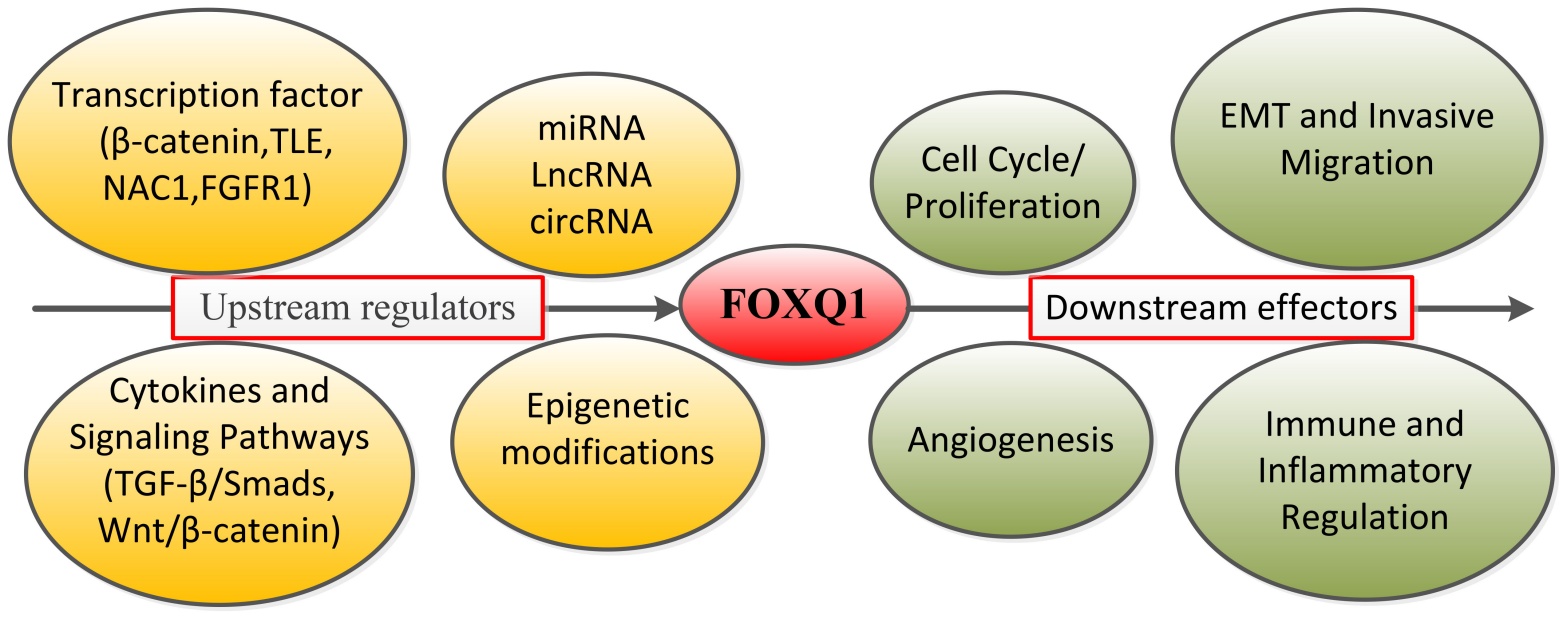
Summary diagram of FOXQ1’s upstream core regulatory factors and downstream effects.

Post-transcriptional regulation of FOXQ1: In the above review of multiple cancers, we have described multiple miRNAs targeting FOXQ1. miR-124 inhibits NPC amplification, migration and invasion by regulating FOXQ1 expression through targeted binding to 3’-untranslated region (3’UTR) (94). Similarly, members of miR-422a, miR-506 and miR-1271 have been found to regulate the expression and biological functions of FOXQ1 (71, 95, 96). Similarly, long noncoding RNA-activated FOXQ1 epitopes promote bladder cancer cell expansion and migration (97). Qi Zhao et al. found that FOXQ1 expression was regulated by Ubiquitin-specific protease10 deubiquitylation, which was effective in alleviating inflammation and apoptosis in acute kidney injury through the CREB5/NF-κB signaling axis (86). In contrast, Ubiquitin-specific protease21 ubiquitination inhibits FOXQ1 expression promoting Bladder cancer EMT and stemness (98). Additionally, FOXQ1 exerts a protective effect against S-AKI-induced inflammation and apoptosis by targeting the CREB5/NF-κB pathway through deubiquitination mediated by USP10 (86). Natalie Atyeo et al. (99) reported that histone acetylation regulating FOXQ1 plays a key role in oral epithelial cell infection. Additionally, recent studies have reported that P300-mediated acetylation of the FOXQ1 complex activates super-enhancers, thereby promoting proliferation and metastasis in CRC (100). FOXQ1 expression is subject to promoter region methylation which plays a key role in the pathological progression of mastocytosis patients (101). Therefore, based on the content of the above review, we summarize the core upstream regulatory factors and downstream effects of FOXQ1 (**Figure 2**).

Inhibitors: We searched the literature and found no direct inhibitors of FOXQ1, but indirect inhibitors have been reported. KH-3 is an effective inhibitor of the RNA-binding protein HuR. By interfering with HuR-FOXQ1 mRNA interactions, KH-3 inhibits breast cancer cell invasion and delays lung colony formation (32). Yunfan Luo et al. (49) reported that FOXQ1 promotes EGFR expression at both mRNA and protein levels. EGFR inhibitor drugs can suppress FOXQ1-induced vasculogenic mimicry formation, thereby inhibiting the growth and metastasis of NPC. Therefore, depending on the underlying pathological mechanism, FOXQ1 can serve as a direct or indirect therapeutic target.

## Mechanism of tissue-specific function

Transcription of FOXQ1 is differentially regulated across different cell types, potentially yielding protein isoforms with distinct or even opposing functions. Kaneda et al. found that FOXQ1 is highly expressed in CRC, promoting tumor formation, growth, angiogenesis, and anti-apoptosis, suggesting that FOXQ1 may play a positive role in certain cancer types (102). FOXQ1 expression was detected in various tumor cells, where it promotes cell growth/proliferation, migration/invasion, angiogenesis, tumorigenicity, and metastasis by upregulating axonal protein 3, zinc finger protein E-box binding homolog 1/2, vascular endothelial growth factor, Wnt proteins, and Bcl proteins. These tumors include breast cancer, liver cancer, glioma, CRC, and ovarian cancer (37, 85, 102, 103).

Mikhail Nikiforov et al. (75, 76) investigated the key role played by the protein FOXQ1 in the development of melanoma, a distinct type of cancer that originates from different cell types compared to other cancers. The transcription factor FOXQ1 suppresses the progression of the same process in melanoma cells that induce carcinogenesis, a process dependent on the balance between two types of proteins: β-catenin and members of the TLE family. When interacting with FOXQ1, these proteins can convert each other into either transcription activators or repressors, thereby inducing or suppressing the expression of N-cadherin (CDH2 gene), a key regulator of tumor invasion and metastasis.

## Conclusion

FOXQ1 has been demonstrated to be closely associated with the development of various of tumors. It orchestrates a wide range of biological functions in human diseases, such as cell invasion and migration, proliferation, differentiation, apoptosis, inflammation production and vascular proliferation, induction of fibrosis and aggressive behavior. Further in-depth studies are required to elucidate FOXQ1 gene expression, protein function, and its mechanisms of action in human disease. Although FOXQ1 has been validated as an oncogene, its downstream targets and the signaling pathways it modulates are governed in a cancer-type-specific manner. Its specific mechanism in tumor infiltration and migration is also not fully understood. Thus, future in-depth studies on FOXQ1 functions may confirm its potential as a key marker of cancer diagnosis and therapy in the future. Future research should focus on developing targeted therapeutic strategies for FOXQ1, such as designing small-molecule inhibitors or RNA therapeutics to precisely regulate its activity. Concurrently, leveraging emerging disease models like organoids and single-cell sequencing will enable systematic analysis of FOXQ1’s spatiotemporal expression patterns and regulatory networks across diverse tissue contexts. This approach will provide actionable biomarkers and therapeutic options for clinical translation.
